# Ten years of NSR—progress and perspectives

**DOI:** 10.1093/nsr/nwae002

**Published:** 2024-01-11

**Authors:** Chunli Bai, Mu-ming Poo

**Affiliations:** Chinese Academy of Sciences, China; Chinese Academy of Sciences, China

This issue of National Science Review (NSR) marks the 10th anniversary of this journal. Within a relatively short period of ten years, NSR has grown into a full-fledged journal with a high international standing and published many high-quality papers that are well received by the international community. At its inauguration in 2014, NSR aimed to be a broad-spectrum journal devoted to publication of high-quality papers in six major scientific areas, including physics & mathematics, chemistry, life sciences, earth sciences, materials science, and information sciences. We also wished to establish a platform for highlighting the latest scientific discoveries and technological development in China and around the globe.

In 2014, NSR began with the publication of review articles, perspectives, research highlights, forums on topical issues, and interviews of prominent scientists. Most issues also include a collection of articles on a special topic, focusing on an active area in science and technology. The publication scope was expanded to include original research articles in 2017, an important step that had greatly enlarged the authorship and readership of NSR. In addition to interviews and forums, other forms of news articles such as featured institutions and news reports have also been published. To cope with the increasing submission, NSR also changed from quarterly to monthly issues. For timeliness of publication, accepted papers could be visited immediately on-line prior to the publication of the printed version. The special topic section, which now includes reviews, original research articles and interviews, are particularly notable, because it provides a useful glimpse on recent progress in a specific field. Bound reprints of special topic articles are distributed at the NSR exhibition booth in major scientific meetings and are well-appreciated by the community.

The past decade has witnessed the rapid progress of science and technology around the globe and the extraordinary growth of Chinese science, as reflected in the number of active scientists and the quality of their scientific output. This is also evidenced by the steady growth of submissions and publication of original research papers in NSR. To cope with such growth, we have established, in addition to editorial boards, special editorial groups that include 130 prominent scientists in China and abroad. As a window for showcasing the progress of Chinese science, publication of more extensive and timely news reports on major scientific achievements in China will be an important mission for NSR in coming years.

There is increasing demand and competition among major scientific journals for publishing the most significant findings in various fields. The desire of most scientists to publish their work in ‘top journals’ (based on impact factors) notwithstanding, we noted that even the most original and innovative findings may likely face strong disagreement of some referees. We encourage the NSR editorial groups to make a special effort to identify manuscripts that are worthy of publication despite the dissenting views of some referees. In 2017, NSR established a Critique and Debate section to provide a platform for scientists to express their opposing views on scientific publications in NSR or other journals. Open debate on scientific findings and interpretations has become rare in major scientific journals, and this NSR section could remedy this situation.

International scientific journals are the main channels of scientific communication around the globe. We are happy to see that geopolitical conflict and economic competition had not affected the international spirit of scientific journals. Globalization is a visible trend among leading scientific publishing enterprises, as evidenced by the establishment of permanent offices in China by major international journals. In the coming decade, NSR aims to become more international in the authorship and readership. In line with the notion that scientific outputs must benefit global societies at large, NSR will also focus on timely publication of research findings that meet the increasingly pressing needs of the world, in areas such as green energy, environmental protection, information technology, sustainable development and human health and diseases.

At this moment of celebration and reflection, we wish to express our gratitude to a large number of scientists who had submitted their manuscripts to NSR, members of six editorial boards and editorial groups who monitored the review process, the invited reviewers around the globe who devoted their precious time for NSR, and the dedicated staff in the editorial office who keep NSR publication on track despite the increasing workload. Together, we all look forward to further growth and prosperity of NSR in the coming decade.

